# Narrative review of current neuromodulation modalities for spinal cord injury

**DOI:** 10.3389/fpain.2023.1143405

**Published:** 2023-03-09

**Authors:** Roi Medina, Alison Ho, Rajiv Reddy, Jeffrey Chen, Joel Castellanos

**Affiliations:** ^1^ Department of Physical Medicine and Rehabilitation, Lake Erie College of Osteopathic Medicine-Bradenton, Bradenton, FL, United States; ^2^ Department of Physical Medicine and Rehabilitation, Baylor University Medical Center, Dallas, TX, United States; ^3^ UC San Diego Health, University of California San Diego, La Jolla, CA, United States

**Keywords:** spinal cord stimulation (SCS), spinal cord injury (SCI), neuromodulation, chronic pain, complete spinal cord injury, incomplete spinal cord injury, transcranial direct current simulation, transcranial magenetic stimulation (TMS)

## Abstract

Neuromodulation is a developing field of medicine that includes a vast array of minimally invasive and non-invasive therapies including transcranial magnetic stimulation (TMS), transcranial direct current stimulation (tDCS), vagus nerve stimulation (VNS), peripheral nerve stimulation, and spinal cord stimulation (SCS). Although the current literature surrounding the use of neuromodulation in managing chronic pain is abundant, there is an insufficient amount of evidence specifically regarding neuromodulation in patients with spinal cord injury (SCI). Given the pain and functional deficits that these patients face, that are not amenable to other forms conservative therapy, the purpose of this narrative review is to examine and assess the use of various neuromodulation modalities to manage pain and restore function in the SCI population. Currently, high-frequency spinal cord stimulation (HF-SCS) and burst spinal cord stimulation (B-SCS) have been shown to have the most promising effect in improving pain intensity and frequency. Additionally, dorsal root ganglion stimulation (DRG-S) and TMS have been shown to effectively increase motor responses and improve limb strength. Although these modalities carry the potential to enhance overall functionality and improve a patient's degree of disability, there is a lack of long-term, randomized-controlled trials in the current space. Additional research is warranted to further support the clinical use of these emerging modalities to provide improved pain management, increased level of function, and ultimately an overall better quality of life in the SCI population.

## Introduction

Neuromodulation is a developing field of medicine that includes a vast array of minimally invasive and non-invasive therapies including transcranial magnetic stimulation (TMS), transcranial direct current stimulation (tDCS), vagus nerve stimulation (VNS), peripheral nerve stimulation, and spinal cord stimulation (SCS). SCS, the most common implantable neuromodulation therapy, is a well-established technique used to reduce the intensity, frequency, and duration of pain that is not amenable to other forms of conservative therapy. First used for the treatment of pain in 1967 by Shealy et al., SCS delivers electrical pulses to nerves along the spinal column through epidural electrodes, which modifies nerve activity and minimizes the sensation of pain reaching the brain (1). The basis of SCS was provided in 1965 by Melzak and Wall, who developed the gate control theory of pain and highlighted the importance of the dorsal columns in treating intractable pain (2). Indications for the use of SCS include pain from Failed Back Surgery Syndrome (FBSS), Complex Regional Pain Syndrome (CRPS), critical limb ischemia, and intractable angina (3–6).

Spinal Cord Injury (SCI) in the United States population has an estimated incidence of 17,810 new cases annually with an estimated prevalence ranging from 250,000 to 368,000 persons (7). SCI can lead to loss of motor and sensory function, neurogenic bowel and bladder, spasticity, and chronic pain. Chronic pain occurs in close to 70% of patients who have suffered a SCI (8). Chronic pain stemming from SCI can be categorized into three main types: nociceptive, neuropathic, and visceral (8). Visceral pain is located primarily in the thorax and abdomen and related to neurogenic bowel and bladder. Nociceptive pain is the most common and can originate from the initial trauma, muscle weakness, spasms, contractures, or overuse. Neuropathic pain after SCI is classified as pain at the level of injury or below and can occur acutely or as late as one year after initial injury (8). Recently, there has been an increased focus on management of neuropathic pain following SCI. Pathophysiology of chronic neuropathic pain following SCI differs depending on whether the neurons involved are located above or below the level of injury. Above the level of injury, hyperexcitability of neurons are thought to be caused by changes in expression of N-methyl-d-aspartate and glutamate receptors, sodium and calcium channels, increased glial activation, and decreasing functional endogenous inhibitory neurons (8). Below the level of injury, the mechanism is not clearly understood but it is postulated that it originates from a more sensitized spinothalamic tract, spontaneous activity in disinhibited polysynaptic pathways, thalamus, and cortex (8). Neuropathic pain secondary to diseases and injuries to the nervous system including SCI have also been treated with SCS (9, 10). Electrical pulses delivered by SCS have stimulation parameters including amplitude, frequency, and pulse width that can be altered to optimize the level of pain relief that is achieved (11, 12).

## Methods

The available literature on the use of SCS, TMS, tDCS, VNS, TENS, and PENS within the SCI population was reviewed. Data resources included relevant literature published from 1965 through April of 2022 that were identified through searches of PubMed, Embase, Cochrane Library, and manual searches of the bibliographies of known primary and review articles. The search strategy focused on patients in the SCI population who were suffering from pain and/or motor deficits. The search terms included “spinal cord stimulation” or “transcranial magnetic stimulation” or “transcranial direct current stimulation” or “vagus nerve stimulation” or “transcutaneous electrical nerve stimulation” or “percutaneous electrical nerve stimulation” in SCI patients. The review focused on randomized controlled trials, systematic reviews, and case reports for pain relief, improvement in motor function, and other outcome measures.

## Modalities

### Paresthesia-based spinal cord stimulation

Paresthesia-based SCS (P-SCS) was the first paradigm developed for use in pain management and involves the application of electrical impulses at a frequency of 30–80 Hz until a non-painful paresthesia is experienced in the targeted dermatomal distribution (13, 14). P-SCS has been studied the most out of all paradigms and many clinical studies assessing efficacy of SCS is based on P-SCS. The proposed mechanism involves the antidromic activation of dorsal horn Aß fibers, which contributes to the activation of inhibitory interneurons that lead to an alteration of projection neuron firing by suppressing the activity of wide dynamic range dorsal horn projecting neurons (15). There is a clear association between the activation of inhibitory interneurons and the presence of neurotransmitters involved in antinociception (16) but the origin of the initial activation is unclear and may not be limited to the activation of the dorsal horn. Other postulated mechanisms of P-SCS include modulation of gliosis and neuroinflammation, as well as orthodromic activation of the dorsal column that leads to activation of serotonergic and noradrenergic descending activating systems with subsequent neurotransmitter release (15, 17).

The use of P-SCS for treating SCI has been investigated and previous studies have demonstrated potential efficacy of this paradigm when addressing pain and other manifestations of SCI. Additional therapeutic actions of P-SCS outside of nociceptive pain control include, but are not limited to, attenuating spasticity (9, 18), regaining motor function (9), regulating blood pressure (19), improving bladder voiding function (20), managing visceral pain (21), and restoring cough (22–25). Studies of pain control in SCI patients have been carried out since the 1970s and early reports of satisfactory pain control were encouraging (26–29). However, other studies have suggested the efficacy of P-SCS for SCI pain to be inferior compared to other interventions and questions regarding long-term outcomes suggest the need for further investigation. Cioni et al. followed a group of 25 SCI patients who underwent SCS and found an initial success rate, defined as the number of patients with greater than 50% pain relief, of 40.9%. However, at 3-year follow up, the success rate decreased to 18.2% (30). A review study from 2009 analyzing 27 published SCS clinical studies revealed a 30%–40% success rate, leading the authors to arrive at a conclusion that there is insufficient amount of evidence for SCS as an indication for pain control in SCI patients (31). An additional study found greater improvement in pain scores with SCI patients that took opioid medications or completed physical therapy when compared to patients that underwent SCS (32). While P-SCS has been the traditional approach of addressing pain through SCS, advancements in the field and increased efforts to improve patient outcomes have led to a vast array of alternative programming options and stimulation settings. These alternative programming options include: High Frequency-SCS (HF-SCS), Burst SCS (B-SCS), Dorsal Root Ganglion Stimulation (DRG-S), Differential Target Multiplexed SCS (DTM-SCS), TMS, tDCS, VNS, TENS, and PENS.

### High-frequency spinal cord stimulation

High-Frequency Spinal Cord Stimulation (HF-SCS) is a paradigm that has emerged and is a broad term used to refer to SCS utilizing electrical impulses at a higher frequency than that used in P-SCS. A frequency of 10 kHz is the most commonly used delivery strategy but there is evidence of efficacy at lower frequencies (33–35). HF-SCS is often used to induce pain control in patients who were unsuccessful with P-SCS and preclinical studies have shown it to have equal to or greater efficacy than P-SCS (36, 37). Compared to the immediate antinociceptive effect of P-SCS, preclinical and clinical data show that pain inhibition induced by HF-SCS has a delayed onset (38). This finding, combined with the understanding that HF-SCS is not dependent on generating paresthesia that overlaps the patient's painful dermatome, suggests that the there is a novel mechanism of action that differs from that of P-SCS (39). In rat models it was shown that HF-SCS was unable to elicit action potentials in dorsal horn nuclei, an essential part of the proposed mechanism behind P-SCS (37). There are “working hypotheses” for the proposed mechanism behind HF-SCS and they include: reversible depolarization blockade, desynchronization of neural signals, membrane integration, and glial-neural interaction (13). Another potential neural mechanism is the generation of an electrical field in the superficial dorsal column that does not elicit an action potential but still causes changes in neuronal excitability and nociceptive transmission in the superficial dorsal horn (38).

Currently, there is limited clinical research assessing the use of HF-SCS on SCI patients, specifically for SCI pain. Preclinical studies have been promising but also scarce when assessing HF-SCS for SCI. A study of two, C-2 transected dogs showed successful recruitment of inspiratory muscles after application of 300 Hz HF-SCS (40). The authors present the HF-SCS method used in the study as a potential alternative method of restoring ventilation in ventilator-dependent SCI patients. In another canine animal model, Kowalski et al. found that HF-SCS to the lower thoracic spinal cord resulted in a generation of airway pressures typical of a normal cough. Mean positive pressure generated was highest at 500 Hz and found to be comparable with those generated from P-SCS (41). With clinical studies, it has been shown in 2 different randomized-controlled trials that HF-SCS is more efficacious than P-SCS when treating chronic back or leg pain (39, 42). However, in both studies, it is not stated that chronic pain experienced by the patients originated from previous SCI. The inclusion criteria in both studies merely mentions “chronic intractable pain of the trunk and/or limbs, refractory to conservative therapy for 3 months”. Other studies of HF-SCS have examined the effect of varying frequencies on pain control, yet pain is either unspecified or of different cause like Failed Back Surgery Syndrome (43, 44). Yamada et al. presents a case report on a 69-year old male with several bilateral upper limb pain from a C4 SCI who underwent 1-kHz HF-SCS (45). One month after implantation, the patient experienced improvement in pain intensity, sleep, and quality of life. At 12-month follow up, there was no increase in pain severity. A retrospective study examining HF-SCS in patients with neurogenic bladder incontinence secondary to SCI or underlying neurologic disease showed promising results as well. All 5 patients in the study had positive outcomes, with episodes of leakage per day improving by an average of 83% (46). Improvement in other parameters such as residual volume and quality of life were also observed. Given the lack of clinical evidence with HF-SCS for SCI, further investigation is necessary before translation into a clinical setting.

### Burst spinal cord stimulation

Burst Spinal Cord Stimulation (B-SCS) is another paradigm that uses bursts of electrical pulses of stimulation as opposed to the continuous pulse used in P-SCS. BurstDR SCS technology consists of bursts of 5 pulses delivered at 500 Hz with a pulse width of 1 msec and inter-burst frequency of 40 Hz, followed by a quiescent period (47, 48). B-SCS has been shown to provide pain relief without relying on paresthesia in a majority of patients and several studies report it to decrease neuropathic pain better than P-SCS and HF-SCS (49–52). The mechanism of action behind B-SCS remains to be fully elucidated. In contrast to P-SCS, B-SCS does not increase activity in the dorsal column nuclei and has no significant impact on gracile nucleus firing (49). This may explain the absence of paresthesia in B-SCS, as the gracile nucleus is the sensory area responsible for information ascending from the dorsal column. Further difference in the mechanism between B-SCS and P-SCS was highlighted by Crosby et al. in a study of rats where it was suggested that unlike P-SCS, B-SCS does not inhibit neuronal excitability of wide dynamic range neurons through GABAergic transmission (53). De Ridder et al. proposed that the mechanism behind B-SCS involves modulation of low-threshold tactile c-fibers, as well as multiplexing which could contribute to modulation of cortical attentional mechanisms (54). Different firing patterns transmit different types of information about a single stimulus and may be involved in different pathways of the spinothalamic tract, such as tonic stimulation firing in the lateral pain pathway and burst stimulation firing in the lateral and medial pain pathway (47, 55). The medial pain pathway has been shown to be responsible for the emotional components of pain, suggesting that B-SCS may play a larger role in improving the behavioral and memory aspect of pain compared to P-SCS (56).

Similar to HF-SCS, the current literature on the use of B-SCS in the SCI population is sparse and mainly consists of case studies. The first B-SCS study for pain was published in 2010 in patients with primarily failed back surgery syndrome and failed neck surgery syndrome (57). Since then there has been additional research for the use of B-SCS for pain, including the SUNBURST trial, which is the largest randomized control trial to date comparing B-SCS to P-SCS (50). In this study, not only did B-SCS demonstrate superiority compared to P-SCS when examining reduction in pain intensity but the authors found that patients preferred B-SCS over P-SCS. A possible mechanism behind this finding may be explained by a SUNBURST sub-study that utilized neuroimaging and found that both the medial and lateral pain pathways were modulated in the B-SCS treatment group, compared to only the lateral pain pathway being affected in P-SCS (58). Three recent case reports highlight the efficacy of B-SCS for SCI pain. One case report described a 53-year old paraplegic female who had been suffering from a two-year history of chronic neuropathic pain in both lower extremities secondary to complete SCI below T5 (59). She experienced both reduction in pain frequency and intensity after B-SCS, with effects lasting at 3-month follow up. B-SCS was also utilized in a 52-year old male diagnosed with at-level SCI neuropathic pain having a T12 neurological level of injury with pain localizing to his right anterolateral thigh (60). Pain intensity and frequency were found to be reduced by half with undiminished efficacy of stimulation at 1-year follow up. A final case report describes a 59-year old male with refractory, bilateral upper extremity pain for 9 years after SCI. The patient initially underwent a 2-week trial that involved both tonic and burst stimulation, where the patient experienced better reduction in pain intensity and less discomfort with B-SCS. After permanent implantation, he experienced a reduction in pain intensity and frequency and also noted improvement in depression and other psychological symptoms that were initially reported (61). The current literature highlights the potential use of B-SCS in the SCI population but further investigation with randomized controlled trials and long-term follow-up periods may be necessary before definitive conclusions are formed.

### Dorsal root ganglion stimulation

Dorsal Root Ganglion Stimulation (DRG-S) is an emerging modality that involves implanting stimulation leads epidurally near one or more dorsal root ganglions (DRGs), which contain cell bodies of primary sensory neurons (PSNs) that innervate specific dermatomes of the patient's body. First described as a modality in 1998, DRG-S has become more prevalent in the current literature with increasing reports showing the potential applicability in the management of chronic pain (62, 63). More traditional modalities, including P-SCS, HF-SCS, and B-SCS, have limitations due to the anatomic location of the stimulation leads within the spine and the delivery of the stimulation (64, 65). As previously mentioned, the leads with traditional modalities are placed in the posterior epidural space, which allow for delivery of stimulation currents directly to the posterior spinal cord tracts. In contrast, DRG-S directly targets the sensory neurons within the DRG in the peripheral nervous system, leading to greater flexibility of the stimulation leads and increased versatility in its usage (65). This allows DRG-S to provide greater stimulation specificity for painful areas and more easily provide pain relief in patients who have specific conditions that are more difficult to treat with traditional techniques, such as distal limb pain and mononeuropathies (63, 66). Proposed mechanism of action through an *in vitro* animal study involves DRG-S demonstrating an alteration in Ca^2+^ influx-slowed nerve conduction velocity, reduced action potential propagation, and neuronal excitability (67). Another proposed mechanism is related to the ectopic activity within nociceptive neurons. It has been shown that there is a profound increase in ectopic activity within the DRG following nerve injury, which can result in the action potentials propagating to the central nervous system (CNS) and ultimately causing neuropathic pain (68, 69). DRG-S may provide analgesia through suppressing neuronal excitability and decreasing ectopic activity of these nociceptive neurons. These mechanisms are further supported by Kent et al. in a computational modeling analysis that examined DRG-S in pain suppression (70).

There are several recent studies demonstrating the efficacy of DRG-S in humans. The first randomized control trial of DRG-S was conducted in patients with CRPS and causalgia of the lower extremities, where DRG-S was demonstrated to be superior to traditional SCS at providing sustained pain relief at 3- and 12-month follow-up (66). Compared to traditional, tonic SCS, DRG-S was also shown to provide greater stimulation specificity for the associated painful areas, less variation in stimulation intensity with postural changes, and greater improvements in functional status, mood, and quality of life (66, 71). In the SCI population, multiple case reports highlight the potential for use of DRG-S in addressing the various manifestations after SCI. Soloukey et al. presents a case series of five motor complete SCI patients who underwent bilateral L4 DRG-S with the aim of evoking two types of muscle responses in the upper leg muscles (72). On the first day DRG-S was applied, all five patients in the study were shown to evoke significant “dynamic” motor response, which is characterized by a clear alternation between contraction and relaxation, and “isotonic” muscle response, characterized by continuous contraction with stable clonus and no visible relaxation. These motor responses were found to be both reproducible and sufficient enough for assisted weight-bearing. Dombovy-Johnson et al. reports on a patient with T11 American Spinal Injury Association (ASIA) B SCI suffering from CRPS predominantly in the left lower extremity (73). After an ineffective four-day trial of P-SCS, the patient experienced a successful seven-day trial of DRG-S with leads placed on the left L4, L5, and S1 DRG. At 6-month follow up, the patient continued to experience a significant decrease in swelling and dysesthesia, as well as improved sitting tolerance. The efficacy of attenuating spasticity after SCI has also been examined with DRG-S in a case study involving a 48-year old male with a 25-year history of T8 motor complete SCI (74). After a history of unsuccessful treatment with oral baclofen, the patient underwent bilateral L2 DRG-S for a five day period. The patient experienced decreased frequency and severity of spasticity, with complete resolution of symptoms in the post-stimulation period beginning on day six until day 13, when symptoms returned. The details of this case study, along with additional studies highlighting the successful use of spinal cord stimulation in patients with SCI, are presented in Table 1. Although early studies of DRG-S for SCI demonstrate adequate efficacy, more long-term studies are warranted for addressing the many manifestations in SCI patients.

**Table 1:** Summary of case reports investigating spinal cord stimulation in patients with spinal cord injury.

Authors	SCI level	Lead placement	Programming parameters	Outcomes
Yamada et al (45)	C4 ASIA A	T1-T3	1-kHz frequency; 90-µs pulse width (HF-SCS)	≥50% pain reduction at 1- and 12-month follow-up per numerical rating scale (NRS; 0–10) Significantly improved sleep and quality of life per Athens Insomnia Scale (AIS) and Pain Self-Efficacy Questionnaire (PSEQ)
Reck & Landmann (59)	T5 ASIA A	T11-L1	40 Hz burst-frequency; 500 Hz intra-burst-frequency; 1,000 mcs pulse duration (B-SCS)	≥50% pain reduction at 3-month follow-up per NRS
Yoon & Kim (60)	T12 ASIA A	T9	40 Hz burst-frequency; 500 Hz intra-burst-frequency; 1 ms pulse width (B-SCS)	≥50% pain reduction at 1-year follow-up per visual analog scale (VAS; 0–10)
Lee et al (61)	C4 (ASIA level unspecified)	C4-C7	40 Hz burst-frequency; 500 Hz intra-burst-frequency; 1,000 ms pulse width; 0.2 mA amplitude (B-SCS)	≥50% pain reduction at 1-month follow-up per NRS
Dombovy-Johnson et al (73)	T11 ASIA B	L4-S1	Unspecified (DRG-S)	Significant decrease in swelling, dysesthesias, and improved sitting tolerance at 6-month follow-up
Soloukey et al (74)	T8 ASIA B	L2	4 Hz frequency; 0.1 mA amplitude; 1000-µs pulse width (DRG-S)	≥50% reduction in severity and frequency of spasticity at day 12 per NRS and Penn Spasm Frequency Scale (PSFS) ≥50% reduction in low back pain at day 12 per NRS Modified Ashworth Scale (MAS) 0 for knee flexors, knee extensors, dorsal ankle flexors, plantar flexors, and hip adductors from Day 1 to Day 13

### Differential target multiplexed spinal cord stimulation

Differential Target Multiplexed Spinal Cord Stimulation (DTM-SCS) represents the latest advancement in SCS and involves simultaneous delivery of multiplexed stimulation patterns to multiple targets within the dorsal column of the spinal cord. DTM-SCS utilizes concurrent electrical impulses that can differ from one another in frequency, amplitude, pulse width, and charge balancing, giving patients greater flexibility and versatility in achieving optimal pain relief (75). It is hypothesized that DTM-SCS provides analgesia through rebalancing interactions between neurons and glial cells that have been affected by the underlying establishment of pain (76). This rebalancing has been demonstrated in animal models to be accomplished through alteration in neuroinflammation and modulation in the expression of proteins involved in ion transport (76, 77). In preclinical studies, DTM-SCS more effectively returned glial and neuronal gene expression back towards pre-pathologic levels compared to high and low-rate SCS (78, 79). Further support favoring DTM over traditional paradigms utilizing single electrical signals is presented by Cedeño et al., where the authors found DTM-based programs provided better relief of pain-like behavior in rodent and ovine models (80).

There is established benefit from the use of DTM-SCS in low back and leg pain, which is highlighted by a 12-month randomized control trial comparing DTM-SCS and traditional SCS (75). Outside of this study, there are limited clinical studies assessing the use of DTM-SCS for pain management. Currently, there is no published research examining the use of DTM-SCS in SCI patients. Given the supporting evidence provided by recent preclinical trials, further research for DTM-SCS use in chronic pain and other manifestations of SCI is necessary. As the treatment landscape for pain management continues to shift towards SCS, integration of novel SCS paradigms can help further improve clinical outcomes.

### Transcranial magnetic stimulation

Non-invasive CNS stimulation modalities that have been studied for use in SCI patients include TMS and tDCS. Magnetic stimulation has been shown to activate motor pathways by directly acting on neurons in the corticospinal tract, causing an initial D-wave and subsequent I-wave (81). In contrast, electrical stimulation through tDCS acts transsynaptically, resulting in I waves without D waves. This difference in mechanism of motor pathway activation between TMD and tDCS may elucidate why muscle responses to magnetic stimulation show longer onset latency, simpler waveforms, and shorter duration with larger amplitudes in comparison to electrical stimulation (81). TMS has many clinical indications including epilepsy, stroke, depression, ataxic disorders, cranial nerve disorders, as well as SCI (81–84). For SCI, repetitive Transcranial Magnetic Stimulation (rTMS) is commonly used and involves the repetitive delivery of magnetic pulses over cortical sites to increase or decrease cortical excitability (85, 86). The major effect of rTMS on the CNS includes changes in neuronal plasticity through long-term potentiation and depression. Other mechanisms contributing to CNS plasticity include, but are not limited to, changes in network excitability, feedback loop activation, and activity dependent metaplasticity (87–89).

Belci et al. conducted one of the first studies examining rTMS in four chronic tetraplegic patients and demonstrated significant improvement in ASIA sensory and motor scores after five sessions of rTMS over a therapeutical motor cortex target (90). An additional randomized, double-blinded study of 17 ASIA D SCI patients receiving 15 daily sessions of rTMS showed similar results. Significant improvement in lower extremity motor score, as well as other parameters assessing gait and spasticity, was observed in the rTMS group compared to the sham group (91). These improvements were seen after the last of 15 rTMS sessions and maintained for two weeks after treatment. A follow up study by Kumru et al. in incomplete SCI patients also found a greater improvement in upper and lower extremity limb strength in the rTMS group compared to the sham control group (92). Although a significant difference was not found in several parameters assessing gait, 71% of the subjects at follow up after rTMS and 40% of the subjects after sham rTMS were capable of performing the ten meters walking test (92). In both studies by Kumru et al. (91, 92) mentioned above, it is important to note that the protocol included the use of rTMS combined with supervised gait training. Kuppuswamy et al. (93) conducted a randomized, sham-controlled cross over trial that revealed no significant differences in ASIA scores between real and sham rTMS. The protocol involved 5 consecutive days of real or sham rTMS in 15 patients with incomplete SCI T1 or above, with a two-week washout period separating the interventions. More recently, Krogh et al. published a randomized controlled trial involving 20 SCI patients who received either rTMS or sham stimulation for 5 consecutive days a week over a four-week period (94). Stimulation was performed immediately before therapy activities that included twice weekly lower limb resistance training and thrice weekly lower limb physical therapy. Lower extremity motor score (LEMS) assessment, which was performed at admission and within 1 week of discharge, increased significantly for rTMS but not for sham and a greater increase was seen with rTMS compared to sham. Although a statistically significant effect was not observed between the groups, rTMS showed more prominent increases in total leg, knee flexor, and knee extensor maximum voluntary contraction compared to sham. These results, along with results from previous studies, reveal that use of TMS for SCI is promising but inconsistent given the utilization of different parameters and adjuvant therapies that are provided concomitantly. Further research is necessary in order to draw firm conclusions about the efficacy of TMS in SCI patients.

### Transcranial direct current stimulation

Transcranial Direct Current Stimulation (tDCS) involves direct, continuous delivery of low-level electrical currents over the scalp using a paired anode and cathode (95). Like TMS, tDCS has a wide range of clinical indications but an increasing number of studies have been carried out to assess its use following SCI for functional recovery and pain management. It is proposed that anodal tDCS increases the discharge rate of active neurons by hyperpolarizing dendrites and depolarizing the cell body, leading to increased cortical excitability (96). While short-term effect on cortical excitability are associated with these changes in neuronal discharge rates, it is postulated that long-term effects may be due to up or downregulation of membrane receptors that lead to changes in cortical synapse strength (96, 97).

The current literature on the efficacy of tDCS for functional motor recovery following SCI is unclear. Several studies have demonstrated the positive effects of tDCS on both upper and lower extremity motor function (98–101). Cortes et al. used one session of 1 mA, 2 mA, or sham anodal tDCS over the hand primary motor cortex in chronic incomplete SCI patients to examine change in hand grasp (98). Significant improvement in peak speed ratio hand grasp was observed in the 2 mA group. Raithatha et. al (99) examined the use of anodal tDCS paired with robot-assisted gait locomotor training in 15 adults with SCI. 9 participants received 36 sessions of tDCS, while 6 participants received an equal number of sham sessions. They reported a significant improvement in manual muscle testing of the lower extremity in the tDCS group, as well as improvements in other outcome measures assessing gait and balance. Yamaguchi et al. (100) reported significant increase in the number of ankle movements in chronic incomplete SCI patients that received anodal tDCS in combination with patterned electrical stimulation of the common peroneal nerve. A recent randomized, sham-controlled trial examined the effects of tDCS, in combination with robotic training, on gait function assessed by the Walking Index for Spinal Cord Injury II (WISCI-II). 43 incomplete SCI patients underwent 30 sessions of either active or sham tDCS and it was reported that there was a statistically significant difference in percentage of participants that improved after the final session compared to baseline (101). 70.0% of patients in the active tDCS group showed improvement in WISCI-II compared to 33.3% in the sham group. A significant difference was also observed at three-month follow up, where improvement compared to baseline was 68.4% in the tDCS group compared to 35.0% in the sham group. Despite the reported positive outcomes, studies showing no significant effect have also been reported (102, 103). More recently, a meta-analysis of 6 randomized controlled trials revealed a marginal significant pooled effect of active tDCS in motor functionality improvement in comparison to sham tDCS (104). In contrast, no significant pooled effect was reported for motor strength.

The use of tDCS in treating neuropathic pain following SCI has also been examined given the difficulty of treatment with other neuromodulation techniques. The analgesic mechanism behind tDCS is complex and not fully understood but the most widely postulated mechanism involves modulation of spontaneous cortical neuronal activity through polarizing the resting membrane (105). Results regarding the efficacy of tDCS in treating neuropathic pain following SCI are inconsistent as well. Yeh et. al (106) conducted a randomized control trial of 12 participants who received 12 sessions of either sham or real tDCS followed by exercise. Although the group receiving 12 tDCS sessions with exercise had a greater decrease in pain intensity compared to the control group that received exercise alone, results were not significant at 4-week follow up. There was also no significant difference found in the characteristics of experienced pain, brain activity, or quality of life between the two groups at follow up. Inconsistent results are further supported by a recent review of six clinical trials. Although Li et al. found that five out of six studies in the review reported beneficial analgesic effects, they concluded that further evidence is needed to confirm the efficacy of tDCS for SCI-related neuropathic pain given the many factors that influence efficacy including stimulation parameters and individual patient characteristics (107). A more recent meta-analysis by Li et al. suggests that rTMS may be superior to tDCS for treatment of neuropathic pain following SCI (108). Although there have been a relatively large number of studies demonstrating the possible benefits of tDCS on improving motor function and relieving pain following SCI, more large-scale, high-quality trials are necessary to accurately assess its efficacy.

### Vagus nerve stimulation

Vagus nerve stimulation (VNS), through a device similar to a pacemaker, stimulates the vagus nerve through regular electrical pulses that ultimately reach the brain. Although only U.S Food and Drug Administration (FDA)-approved for treatment-resistant epilepsy and depression, recent exploration for its use in post-stroke recovery has laid the foundation for a similar use in the SCI population (109). Clinical findings demonstrate pairing VNS with rehabilitation therapy facilitates synaptic plasticity in spared networks and improvement in post-stroke motor recovery (110, 111). Typical VNS stimulation parameter used for targeted plasticity therapy involves a stimulation train of 0.8 mA, 0.1-msec biphasic pulses at 30 Hz (112). Although clinical studies assessing the efficacy of VNS in SCI patients is limited, pre-clinical studies highlight possible mechanisms and provide promising results. VNS was shown to alleviate neuroinflammation after SCI through promoting the shift of pro-inflammatory M1-polarized microglia to anti-inflammatory M2 phenotype *via* upregulation of alpha 7 nicotinic acetylcholine receptors (113). Specifically, VNS inhibited pro-inflammatory factors such as Tumor Necrosis Factor (TNF)-α, Interleukin (IL)-1β, IL-6 and increased expression of anti-inflammatory factor IL-10. In a rat model, VNS delivered through cuff electrodes implanted 7 weeks after bilateral incomplete contusive C7/C8 SCI in combination with rehabilitation training resulted in better recovery of forelimb function compared to rehabilitation training alone (114). Significant improvement in volitional forearm strength was maintained after cessation of stimulation, indicating possible lasting effects (114). In addition to the trained forelimb motor tasks, VNS paired with rehabilitation training improved similar, but untrained, tasks such as grip strength (114). Given the direct implications of VNS on autonomic tone and the vulnerability of the SCI population to suffer from extreme hypertension in the form of autonomic dysreflexia, as well as orthostatic hypotension, Sachdeva et al. investigated the safety and cardiovascular control of VNS after experimental spinal cord injury (112). Intermittent VNS stimulation showed a transient reduction in heart rate, no change in blood pressure, and did not trigger episodes of autonomic dysreflexia (112). Clinical studies will be important to ascertain the overall effect and efficacy of VNS on motor recovery after SCI but the preclinical studies provide reason to believe there is a space for VNS, in conjunction with rehabilitation therapy, in those with SCI.

### Peripheral nerve stimulation

Transcutaneous electrical nerve stimulation (TENS) is a safe and accessible modality that delivers electrical pulses through the skin to the body to stimulate nerves to relieve pain and treat disease (115). Similar to TENS, functional electrical stimulation (FES) is another form of surface electrical stimulation used to address muscle re-education, spasticity, and functional capacity (116). While TENS is used to stimulate sensory nerves and is primarily used to reduce pain and muscle tone, FES is often times paired with functional activity and primarily stimulates motor nerves to achieve muscle contraction (116). Percutaneous electrical nerve stimulation (PENS) uses acupuncture-like needles positioned over muscles to stimulate peripheral sensory nerves and has advantages of both TENS and electroacupuncture (117). Research investigating the efficacy of PENS in persons with SCI is limited (118), however, TENS and FES are both therapies that have garnered attention in the field of neurorehabilitation. TENS is proposed to provide its analgesic effect through maintenance of spinal opioid receptors through inhibition of mitogen activated protein kinase and proinflammatory expression, activation of glial cells to release inhibitory neurotransmitters, and reduction in dorsal horn neuron activity (119–121). Recent metanalysis involving six randomized-controlled trials and 165 cases revealed favorable results for the use TENS in pain secondary to SCI (122). The difference in VAS between the observation and controlled groups was statistically significant and the short-form McGill pain questionnaire (SF-MPQ) was generally lower in the TENS groups in comparison to the control groups (122). In addition to pain, peripheral nerve stimulation in persons with SCI has also been reported to improve lower extremity muscle mass, spasticity, neurogenic bladder, and respiratory function (116, 123–126). Although peripheral nerve stimulation may be safer and more accessible, the lack of studies with long-term follow up on larger sample sizes indicate implanted devices may provide patients with better long-term results.

## Discussion

Neuromodulation continues to be a topic of interest as a means of treating chronic pain. SCS, TMS, and tDCS continue to gain traction as potential treatment modalities for refractory pain. Recent studies analyzing their efficacy have shown positive preliminary effects. However, the therapeutic efficacy in the SCI population has been poorly studied and largely unmet.

Out of all the current neuromodulation modalities, P-SCS has been the most studied, specifically for the treatment of nociceptive pain in SCI patients. It has also been shown to have other therapeutic actions including attenuating spasticity, regaining motor function, regulating blood pressure, improving bladder voiding function, managing visceral pain, and restoring cough. However, further review studies and other studies analyzing the long-term outcomes of SCS in the SCI population have brought into question the true efficacy of P-SCS in providing adequate pain relief.

HF-SCS and B-SCS have also shown promising results, but limited studies have been completed. Cases studies for the use of HF-SCS in patients with SCI have noted improvement in pain intensity, sleep, and quality of life. Additional retrospective studies in patients with neurogenic bladder incontinence secondary to SCI or other underlying neurologic disease have also shown positive outcomes. Recent case studies regarding B-SCS, have highlighted its efficacy for SCI pain, with patients having a better reduction in pain intensity and frequency with additional noted improvement in psychological symptoms.

Emerging modalities such as DRG-S, TMS, and rTMS have already been demonstrated to show efficacy for managing different conditions. The current clinical indications for DRG-S include managing chronic pain in CRPS, neuropathic pain stemming from phantom limb pain, FBSS, post-herniorrhaphy pain, perineal, and pelvic girdle pain. Recent case series of DRG-S in motor complete SCI patients illustrated the ability to evoke significant and reproducible “dynamic” motor responses that were sufficient enough for weight-bearing. Other case studies have also indicated the efficacy of DRG-S on attenutating spasticity after SCI. TMS has current clinical indications for use in epilepsy, stroke, depression, ataxic disorders, cranial disorders and SCI. In the setting of SCI, rTMS has been commonly used with a recent study showing significant improvement in ASIA sensory and motor scores in four chronic tetraplegic patients. Additional studies have also shown improvement in limb strength and increases in total leg, knee flexor, and knee extensor maximum voluntary contraction. However, these studies all used different parameters with additional adjuvant therapies being provided concomitantly.

There is currently no published research regarding the use of DTM-SCS in SCI patients. Even outside the scope of SCI patients, there is minimal clinical data assessing the use of DTM-SCS for pain management. Similarly, the current literature regarding the efficacy of tDCS for functional motor recovery and for treating neuropathic pain following SCI has been unclear and inconsistent.

## Conclusion

The continued advanced in the field of neuromodulation has led to the development of new treatment paradigms. Recently, the use of neuromodulation to improve pain and function in the SCI population has become a topic of interest. Patients with poorly controlled pain often lose their motivation to be active, perform daily tasks, and participate in therapy. Left untreated, these patients experience a decrease in level of functionality, independence, and quality of life. Currently, HF-SCS and B-SCS have shown the most promising effects with improving pain intensity and frequency. In SCI patients struggling with refractory pain, these two modalities can potentially be used as future first-line treatment options. On the other hand, DRG-S and TMS have shown to increase motor responses, improve limb strength, and even improve ASIA sensory and motor scores. These modalities have the potential to be used first as a means to improve a patient's degree of disability and ability to perform activities of daily living. However, the main limitation regarding these modalities remains the lack of long-term, randomized controlled trials. Further large-scaled trials must be completed in order to determine the clinical efficacy of each method and to determine what additional adjuvant therapies should be provided concomitantly in order to maximize their therapeutic effects. Although early results have been promising, additional research will further support the use of these emerging modalities to hopefully provide improved pain management, increased level of function, and ultimately an overall better quality of life in the SCI population.
